# 1280. Retrospective Chart Review of Diabetic Foot Infection Microbiologic Data at UnityPoint Health Des Moines

**DOI:** 10.1093/ofid/ofad500.1119

**Published:** 2023-11-27

**Authors:** Alexis Clouse, Sydney Blackmer, Nathaniel Holte, Kayla Olstinke, Alivia Passet, Amanda Bushman, Leyla Alexandra Best

**Affiliations:** UnityPoint Health - Iowa Methodist Medical Center, Winterset, Iowa; Drake University and UnityPoint Health Des Moines, Des Moines, Iowa; UnityPoint Iowa Methodist Medical Center, Des Moines, Iowa; Drake University and UnityPoint Health Des Moines, Des Moines, Iowa; Iowa Methodist Medical Center, Des Moines, Iowa; UnityPoint Health Des Moines, Urbandale, Iowa; Unity Point Health, Urbandale, Iowa

## Abstract

**Background:**

Foot infections are a common and serious complication in people with diabetes. Pseudomonas aeruginosa has traditionally been considered a common pathogen in diabetic foot infections (DFI). However, newer data has not found this to be true in clinical practice. In addition, the 2012 Infectious Diseases Society of America guideline for DFI states that “empiric therapy directed at Pseudomonas aeruginosa is usually unnecessary except for patients with risk factors for true infection with this organism”.

**Methods:**

This study is a retrospective chart review of patients presenting with diabetic foot infections at regional teaching hospitals in the Midwest between August 1, 2021 and August 1, 2022. All the patients had either a bone, tissue, or wound culture obtained by podiatric medicine. Descriptive statistics were used to determine relationships between variables and the frequency of P. aeruginosa in culture. The primary objectives of this study were to quantify the prevalence of and determine predictors of pseudomonas DFI.

**Results:**

There were 460 cultures reviewed in the specified time period with 168 cultures meeting criteria for inclusion in the final review. The most commonly isolated organisms were streptococci (29.8%) and methicillin-susceptible Staphylococcus aureus (26.2%). Pseudomonas was isolated in 21 of the 168 cultures reviewed (12.5%), with 17 of them being from non-bone cultures and 3 from bone cultures. The risk for pseudomonas appeared to increase with recurrent or relapsed diabetic foot infections (90.5%), having a previously isolated gram-negative pathogen in the last year (38%), and having a known pseudomonas colonization within one year (33.3%).

**Conclusion:**

Streptococcus and Staphylococcus remain the most common organisms in DFI. Pseudomonas is a less common organism in DFIs; however, our study suggests rates of PSAR may be higher than previous studies have discovered. Risk factors for pseudomonas in our study appear to be recurrent or relapsed diabetic foot infections, having a previously isolated gram-negative pathogen in the last year, and having a known pseudomonas colonization within one year. Though some individualized risk factors can be useful, local epidemiology and resistance patterns remain essential for antibiotic treatment considerations.

**Disclosures:**

**All Authors**: No reported disclosures

